# Implications of the COVID-19 Pandemic on Children and Adolescents: Cognitive and Emotional Representations

**DOI:** 10.3390/children9030359

**Published:** 2022-03-04

**Authors:** Alexandra Maftei, Ioan-Alex Merlici, Iulia-Cristina Roca

**Affiliations:** 1 Department of Educational Sciences, Faculty of Psychology and Education Sciences, Alexandru Ioan Cuza University of Iaşi, 3 Toma Cozma Street, 700554 Iasi, Romania; ioan.alex.merlici@gmail.com; 2 Surgery II Department, Faculty of Medicine, Grigore T. Popa University of Medicine and Pharmacy, 700115 Iasi, Romania; iuliaroca@yahoo.com

**Keywords:** COVID-19 pandemic, children, healthcare, adolescents, emotions, coping mechanisms

## Abstract

The present research investigated children and adolescents’ explicit and spontaneous representation of the COVID-19 pandemic and their related emotions, cognitions, and coping strategies. We explored the self-reported protective factors and coping mechanisms, in addition to similar attributional emotional experiences, i.e., the ways participants evaluated others’ pandemic experiences. Our sample consisted of 155 children and adolescents aged 10 to 13 (*M* = 10.70, *SD* = 0.85, 56.1% females). We designed a 12-item survey and analyzed our data using both qualitative and quantitative methods. Our findings suggested that most children and adolescents associated masks with the thought of the novel coronavirus, and the most frequently associated emotion was sadness (45.2%), followed by fear (17.4%). Generally, participants reported a medium level of perceived adverse effects of the pandemic, mainly because their regular physical school classes moved to the online setting. We also found a significant association between children’s self-reported levels of harmful effects of the pandemic and perceived adverse effects on their families. Most participants expressed their dissatisfaction concerning online school classes, primarily due to poor online interaction. In our sample, the children and adolescents reported positive thoughts and family relationships as their primary coping mechanisms during the pandemic, suggesting similar perceived coping mechanisms in the others around them. Finally, more than half of the participants considered that the COVID-19 pandemic had no positive effects, while 40% considered the increased time spent with their families the primary positive consequences following the COVID-19 health crisis. Results are discussed regarding their implications concerning healthcare, social, and educational policies.

## 1. Introduction

COVID-19 is an infectious disease, first reported in Wuhan, China, on 31 December 2019 [1]. Common symptoms include dry cough, dyspnea, myalgia, fatigue and joint pain, gastrointestinal symptoms, dysgeusia or ageusia (a distortion of tastes or a complete loss of taste), anosmia (a partial or complete loss of the sense of smell), and fever [2]. The first confirmed case of COVID-19 in Romania was reported on 26 February 2020. Out of the first 147 confirmed cases in Romania, 88 were imported, with most carriers (sixty-four) coming from Italy [3]. The first three deaths caused by COVID-19 infections were reported on 22 March 2020. Since then, more than 424 million cases have been reported worldwide (time of writing: February 2022), and more than 5.89 million people have died due to COVID-19.

In Romania, more than 2.2 million people were infected, and over 62.000 have died [4]. At the beginning of the pandemic, in 2020, the country experienced a similar infection rate and patterns as other European countries, but the numbers increased rapidly in 2021 until Romania’s health system was torn apart by the pandemic. The deadly wave emerging in the fall of 2021 pushed Romania to the top of deadly infections with COVID-19, having the highest death rates in Europe (with more than 500 deaths per day).

On 11 March 2020, all public schools in Romania were closed, following a Ministry of Education and Research decision on 9 March 2020 [5]. Although this was initially intended as a short-term measure, establishing the state of emergency in Romania on 16 March led to the schools’ long-term closure, and educational activities resumed in the online environment [6]. The long-term adverse effects of the pandemic on the students’ performance are still investigated; however, it is essential to note that, at the beginning of 2021, approximately 237,000 school and high school students had no internet access, and approximately 287,000 students lacked the equipment needed in order to attend online classes [7]. Furthermore, the high school graduation exam’s passing rate during the summer of 2020 was the lowest in the last six years (72.9%), compared to the rate of 2019, 75.5% [7].

In a study that analyzed the content of public discourse [8], middle school and high school students mentioned the chaotic program and communication, the increased homework, and the difficulties of those with poor material conditions as disadvantages of online education during the pandemic. When considering the advantages, they reported more comfort in studying from home, discovering new hobbies, and more time to prepare for the graduation exams. Ionescu et al. [9] suggested that both students and teachers rarely used the e-learning platforms before the pandemic. Furthermore, their study results that relied on opinion polls indicated that only 17% of the teachers who participated in the study and no high school or middle school students used the e-learning platform before the pandemic. The results also suggested that the e-learning platform was perceived as an effective solution for the pandemic, although it required good collaboration between parents and teachers and constant monitoring of the students’ behavior.

Overall, the access to the resources needed for online learning in Romania was poor. According to the European Center for the Development, the number of children, adolescents, and their families lacking the appropriate resources to access online classes were around 250,000 learners (9% from the total population of learners in State pre-university education), according to the European Centre for the Development of Vocational Training (CEDEFOP) [10]. A similar survey conducted by the Romanian Ministry of Education (2021) stated more than 287,000 Romanian students (children and adolescents) lack the necessary technological devices to access online education [11].

Although it was generally suggested that children and adolescents are usually less vulnerable to severe symptoms or risk of death caused by the COVID-19 disease [12,13], they are still affected both by the virus and the restrictive measures taken by the governments. For example, Duan and their collaborators [14] suggested that residence in urban areas, implementation of precaution measures, being female, and having a family member or friend infected with COVID-19 were associated with increased anxiety levels among adolescents during the pandemic. Similarly, smartphone and internet addiction, residence in urban areas, separation anxiety, and physical injury fear were associated with depressive symptoms. Furthermore, children and adolescents may become asymptomatic carriers and transmit the disease to vulnerable relatives, e.g., their grandparents.

Furthermore, the impact of the COVID-19 pandemic on the mental health of children and adolescents had been the subject of a growing number of studies, most of them highlighting the adverse psychological effects of this health and social crisis, e.g., anxiety, depression, and post-traumatic symptoms [15], as well as stress, worry, and helplessness [16]. Moreover, the systematic review conducted by Meherali and their collaborators [16] highlighted that (1) both children and adolescents are more prone to experience increased rates of depression and anxiety during and after the COVID-19 pandemic, which is a highly concerning fact due to the short and long-term implications; and (2) compared to adults, the long term adverse consequences of the COVID-19 on children’s and adolescents’ mental health are higher.

Given these significant implications of the pandemic, our study aimed to investigate the ways children and adolescents explicitly and spontaneously represent the COVID-19 pandemic and the related emotions, cognitions, and coping strategies. More specifically, we aimed to shape their cognitive and emotional representation of the COVID-19 pandemic. Explicit representations involve the cognitive and neuronal processes that shape the transition from the subconscious (implicit) to verbally reportable (explicit) knowledge, basically indicating awareness for learned associations [17]. Examining children’s and adolescents’ explicit and spontaneous representations gives valuable tools for enhancing adults’ ability to understand their emotional and behavioral reactions towards their social environment, as previously suggested by [18]. Furthermore, examining the self-reported coping strategies used in the COVID-19 would further provide a more comprehensive view of how families and society could address the potentially harmful, long-term effects of similar future social and health crises [19].

The present study is anchored on Social Representations Theory (SRT) [20], a model generally used to understand people’s everyday thinking. SRT also offers a framework for understanding the social strategies that individuals might use when dealing with new risks, such as the COVID-19 pandemic. However, the COVID-19 representations and the general representation of risks are not homogeneous throughout society, given that such crisis moments might generate (different representations among different groups [21]. Furthermore, in the context of highly health-related risky social situations (i.e., the pandemic), emotions play a significant role through emotional anchoring and emotional objectification mechanisms [22]. This is all the more important for children and adolescents, who were found to be more vulnerable than adults in the COVID-19 pandemic context [16].

### 1.1. Children and Adolescents’ Representation of COVID-19 and Perceived Adverse Effects

Several studies investigated children and adolescents’ representation of COVID-19 and perceived adverse psychological effects. For example, Idoiaga Mondragon et al. [23] conducted a qualitative study that reported mixed feelings among the children in their research sample. Even though they reported being happy to spend time with their families, the children also reported worry, fear, loneliness, sadness, nervousness, boredom, and anger. The authors further pointed out that the negative emotions are associated with the restrictive measures and the virus itself since children perceived this new threat as uncertain and unfamiliar. Similarly, Idoiaga Mondragon et al. [23] conducted a free association exercise completed by a sample consisting of children. The content analysis suggested that the children represented COVID-19 as an enemy that the doctors are fighting against.

Furthermore, the main reported reason behind the children’s fear of getting infected was the possibility of infecting their grandparents and, consequently, feeling guilty. Children’s reported emotions included fear, nervousness, loneliness, sadness, boredom, and anger. Additionally, the results from a survey that included participants aged between 15 and 21, both healthy and diagnosed with cancer, suggested the most common perception about COVID-19 was that it is moderately or slightly dangerous. The participants also considered it hazardous for older adults and those with chronic diseases [24].

Considering the decision of several governments to shut down public schools and venues that are usually frequented by the youth (e.g., cinemas, theatres, clubs, amusement parks) and to limit the time spent outside by the citizens, one would intuitively expect that children and adolescents may perceive several negative aspects of the pandemic and the restrictive measures. Ravens-Sieberer et al. [25] reported that in a sample consisting of 1040 participants aged between 11 and 17 and 546 participants aged between 7 and 10, most participants reported feeling burdened by the imposed restrictions; 65% of them reported school and learning as more exhausting than before, and 39% reported a deterioration of their relationships with friends. Furthermore, Orgilés and their collaborators [26] suggested that parents generally reported significant changes in their children’s emotional state during the pandemic, the most common symptoms including difficulties concerning concentrating, boredom, irritability, restlessness, nervousness, loneliness, and uneasiness.

Valadez and their collaborators [27] suggested that adolescents reported being less worried about the pandemic than children. A small effect of gender was also observed, with girls reporting deeper concerns, sympathy, and personal satisfaction. When asked about enjoyable aspects of staying at home, the most common answers were related to spending time with the family or having more time for homework. When they were asked about the unpleasant aspects of staying at home, the participants’ most common answers were related to the inability to go outside or meet their friends and peers and a higher amount of homework [27].

Furthermore, Dunton and their collaborators [28] suggested that, during the early months of the pandemic, parents reported decreases in physical activities, children spending, on average, over eight hours a day engaging in leisure activities that did not involve physical activity. In addition, parents of children aged between 9 and 13 reported higher sedentary behavior than parents of children under 9. Francisco et al. [29] reported that children and adolescents also displayed decreased physical activity and increased screen-time, anxiety-related symptoms and behaviors, and more sleep hours each night during the pandemic. However, having an outdoor space in their homes (e.g., a garden or a balcony) was associated with lower anxiety levels. Finally, in a sample consisting of 1054 Canadian adolescents, the participants reported being worried about their education and peer relationships in particular. COVID-19 related stress was also associated with loneliness and depression, especially for those that spent more time on social media [30].

### 1.2. Emotional Responses during the COVID-19 Pandemic

Children’s and adolescents’ abilities to recognize other people’s emotions are imperative for their development and adaptation to the environment. It was suggested that, as they grow older, children seem to gradually improve their ability to associate a specific context with the appropriate emotion [31] but also recognize specific cues for happiness, fear, surprise, and disgust. However, previous research suggested that recognizing sadness and anger remains relatively constant between 6 and 16. By middle childhood, children may recognize emotions approaching adults [32]. It would also seem that children are more likely to recognize happiness than other basic emotions [33], also more likely to mislabel an ambiguous emotion as happiness. However, they appear to be more likely to recognize subtle hints of sadness than anger. Out of the six basic emotions, disgust appears to be the least recognizable by children [34].

Previous research also suggested the role of the parents in the children’s development of emotion recognition. The results presented by Castro et al. [35] suggested that parents’ beliefs regarding emotions, parents’ emotion labeling and teaching behaviors, and their ability to recognize their own children’s emotions were associated with children’s ability to recognize their parents’ emotions. In addition, de Los Reyes and their collaborators [36] suggested that both adolescents’ and parents’ reduced ability to recognize emotions was associated with a higher level of perceived discrepancies and contradictions in their relations with each other.

To our knowledge, a limited number of studies investigated children’s ability to recognize other individuals’ emotions in the context of the COVID-19 pandemic. A considerable amount of the current studies relied on parents’ reports of their children’s behaviors and emotional states. However, some recent studies suggested potential aspects of the parents’ mental wellbeing that their children might notice and influence them. In one study [37], Italian mothers reported a delay in their children’s sleep timing. The children also displayed more emotional, conduct, and hyperactive symptoms associated with their mothers’ psychological difficulties. In another study [25], 27% of the children and adolescents reported having more arguments with their family, while 37% reported that their arguments usually had with their children escalated more often.

Spinelli et al. [38] reported that the perception of the difficulty of the quarantine predicted both the parents’ and the children’s wellbeing. Furthermore, the quarantine’s impact on children’s emotional and behavioral problems was mediated by parents’ individual and dyadic stress. Finally, the findings suggested by Russell and their collaborators [39] suggested potential spillover effects of parents’ depressive symptoms towards their children, and anxious parents may adopt compensative behaviors to protect children against the adverse effects that the adults feel.

More importantly, the emotional contagion between parents and children generally experienced during the COVID-19 pandemic had been suggested in a growing number of studies [40,41], highlighting the significant role played by parents for their children’s emotion regulation processes [42]. Thus, it is all the more important to explore the way children and adolescents understand and represent the pandemic and the associated emotional patterns for them and the others around them.

### 1.3. Coping with the COVID-19 Pandemic

Previous studies investigating children and adolescents’ coping strategies during the COVID-19 pandemic mostly suggested nonsignificant differences between the two age groups concerning how they cope with isolation and the pandemic’s effects. For example, Orgilés et al. [43] reported that, in a sample that consisted of parents of Italian, Spanish and Portuguese children aged between 3 and 18, the most frequent coping strategies endorsed by children were accepting the situation, collaborating with quarantine social activities, pretending nothing is happening, highlighting advantages of staying at home and not appearing worried about the situation. In addition, Italian children were least likely to manifest worry, and Portuguese children were more likely to use humor. Overall, coping styles oriented towards tasks or avoidance of the situation were better associated with psychological adaptation, while those oriented towards emotions were associated with anxiety symptoms and mood, sleep, and behavior alterations.

Duan and their collaborators [14] reported similar results in a sample of adolescents, where coping styles oriented towards emotions were associated with increased depressive symptoms. In a study conducted by Pigaiani et al. [44], adolescents reported various adaptive coping strategies, including planning their daily routine, engagement in structured activities, development of new interests, and giving a positive reading of the ongoing period. Furthermore, many adolescents shared their feelings with other family members and revaluated their family relationships. However, increased anxiety symptoms and a change in subjective wellbeing were also reported. In another study focusing on adolescents, Cauberghe et al. [45] suggested that loneliness had a more substantial negative impact on adolescents’ happiness than anxiety. Anxious participants were more likely to use social media platforms to cope with their anxiety. Participants who reported feeling lonely used social media platforms to communicate with their relatives and friends. However, this coping strategy had no significant effect on their happiness.

## 2. Method

### 2.1. Study Design and Participants

Our cross-sectional survey sample consisted of 155 children and adolescents aged 10 to 13 (*M* = 10.70, *SD* = 0.85), balanced in gender (56.1% were females). They were all students in a public school in a north-eastern Romanian town. Participation was voluntary, and all answers remained anonymous. Before the study, the researchers sent consent forms to the adolescents’ parents, and almost all parents agreed to participate (86% acceptance rate). Students were informed that they could leave the study at any time, that there were no right or wrong answers, and that all the data and information they shared would remain confidential. The average time needed to answer all questions was about 15 min. All students received colorful stickers for their participation.

The instruments were administered ten months after the COVID-19 pandemic’s outbreak by the school psychologist, a familiar person to the students that formed the current research sample. When students answered the research questions, the school had been closed and re-opened many times since the general lockdown in March 2020. In turn, students returned to school in a hybrid system that allowed them to physically participate two weeks out of four. All participants responded individually, and their answers were private. After they handed out the paper with their answers, participants were debriefed and invited to ask questions about the study and express their thoughts and emotions regarding the research material, i.e., the pandemic and online classes. The study was designed following the Declaration of Helsinki and the national laws from Romania regarding ethical conduct in scientific research. The Ethical Board from the faculty where the authors are affiliated approved the study.

### 2.2. Research Materials

We designed a 12-item survey (see Appendix A) that allowed a deeper exploration into the students’ perception of the pandemic, as well as their related emotions, their own and perceived others’ coping mechanisms, in addition to the way they perceived the online classes they had to attend due to the pandemic. More specifically, we first asked participants to write the first three words that came to their mind when they thought about the COVID-19 pandemic, i.e., “When you think about the COVID-19 pandemic, which are the first three words that come to your mind?” Eiguren and their collaborators [46] used a similar qualitative approach when exploring the social and emotional representations related to the COVID-19 pandemic. As the authors suggested, “this method is based on the premise that words are not independent of each other, but reflect underlying themes”, and “all discourse is expressed from a set of words that constitute units of meaning independently of their syntactic construction” ([46], p. 5). In addition, other research used this approach when exploring the COVID-19 social representations in children and adolescents [47,48,49].

The second question assessed their related emotions: “When you think about the pandemic, which is the emotion you usually feel?”. Again, participants had seven options: six of them mentioned the primary emotions as suggested by Ekman [50], i.e., happiness, sadness, surprise, fear, anger, and disgust, and one of the options was “I don’t know”.

Ekman’s theoretical framework described these emotions as emotions that “(...) evolved for their adaptive value in dealing with fundamental life tasks” [51]. Furthermore, in all cultures, these emotions are recognizable through the same cues [52], although a limited amount of evidence [33] suggests that people may be more likely to recognize the emotions of individuals of the same race. Thus, we used Ekman’s six basic emotions model due to its simplicity and focus on universally recognizable emotions that children also perceive from an early age. Even though children can understand the concept of mixed emotions from an early age [53], they might also have varying abilities in understanding emotions, and some emotions might be easier to identify and label than others. For example, Guarnera et al. [54] reported that happiness was the easiest emotion to identify in a sample that included both adults and children, while disgust was the most difficult. Therefore, we adopted Ekman’s model since those emotions are universally recognizable and easy to identify and label.

Previous research suggested associations between children’s linguistic competence and emotional competence [55]. Notably, previous research [56] also suggested that children are more likely to associate a situation or scenario with an emotional label than a facial expression representing the respective emotion.

The third question assessed participants’ perceived adverse effects of the pandemic: “We often hear about the negative consequences of the current pandemic. However, these adverse effects may not be felt by everyone, or they might feel different for each person. On a scale ranging from 1 (not at all) to 5 (very much), how much did the pandemic negatively affect you?”. Next, participants were asked through an open question to explain their answer to the third question (i.e., “Why?”). Questions 5 and 6 were similar to the third and the fourth, but participants answered these questions by referring to their family: “On a scale ranging from 1 (not at all) to 5 (very much), how much did the pandemic affect your family, negatively?” (Question 5); “Why?” (Question 6).

The next part of the survey addressed participants’ opinion about the online classes they had to attend in the past year: “Due to the pandemic, classes had to be held online, in most of the past few months. On a scale ranging from 1 (not at all) to 5 (very much), how much do you like attending online classes?” (Question 7); “Why?” (Question 8). Next, we investigated participants’ coping strategies considering the pandemic and the way they perceived other people’s resources: “Which are, in your opinion, the things that helped you the most to cope with and get through the changes and the general context brought by the pandemic?” (Question 9); “Which are, in your opinion, the things that helped other people the most to cope with and get through the changes and the general context brought by the pandemic?” (Question 10). The last two questions were designed to assess the perceived potential positive effects of the pandemic: “In your opinion, are there any positive sides or effects of the COVID-19?” (Question 11); “Why?” (Question 12). Finally, we assessed participants’ gender and age.

### 2.3. Results

We analyzed our data using a mixed-method approach. We performed content analyses and frequency analyses to explore the participants’ answers to the open questions (i.e., Questions 1, 4, 6, 8, 9, 10, and 12). For most of the questions, we synthesized all the participants’ answers and coded them into categories after first assessing, through a preliminary investigation, the main correspondences, and differences. In other cases (e.g., the first question), we performed frequency analyses and provided the participants’ exact words without placing them into categories to increase the clarity and accuracy of their explicit representations of the pandemic. We used multiple perspectives for the same purpose related to the quality and clarity of the coding procedure. More specifically, two other researchers (i.e., other than the authors) analyzed and pre-categorized each item’s responses to avoid missing essential results. The two other researchers were part of the same university as two of the authors, specializing in developmental psychology. Each answer and explanation were analyzed and included in broader categories. We then broke the data analytically and focused on in-vivo-coding [57]. Therefore, our approach to qualitative content analysis was inductive. The independent open coding process involved reading each explanation and example offered by the participants and then placing them into categories. Cohen’s kappa (κ > 0.80) indicated strong inter-rater reliability between the coders [58] for each of the seven open item answers. We also used the 24.0 SPSS program to analyze the quantitative data.

A total of 138 answers were offered to the first question (“When you think about the COVID-19 pandemic, which are the first three words that come to your mind?”). Some children did not offer any answers to this question, and most of them only wrote one word. However, most children mentioned the word “mask” as the word that comes to their mind when thinking about the pandemic (*N* = 57; 41.3%; see Table 1). Participants’ answer to the second question suggested that the emotion children and adolescents associated the most with the pandemic were sadness (45.2%), followed by fear (17.4%), an unidentified emotion, “I don’t know”—14.8%, disgust (12.3%), and anger (10.3%).

Most participants reported a medium level of adverse effects of the pandemic upon them, i.e., answers to the question “On a scale ranging from 1 (not at all) to 5 (very much), how much did the pandemic affect you, in a negative way?” (see Table 2). However, higher levels of perceived adverse effects were reported concerning their families. Spearman correlation results suggested a significant association between children’s self-reported levels of harmful effects of the pandemic and perceived adverse effects on their families (*r* = 0.470, *p* < 0.001).

The children and adolescents in our sample explained that the adverse effects they reported were primarily because the classes moved to the online setting (26.37%, i.e., 24 answers out of a total of 91 given), followed by the lack of interaction with their friends (13.27%) and a general feeling of worry (13.98%); see Table 3.

The adverse effects they reported concerning their families were mainly because the participants’ families had to adapt to the online classes imposed by the pandemic context (26.37%), followed by their negative emotional experiences due to the pandemic (13.98%); see Table 4.

Next, participants rated their satisfaction concerning the online classes they had to attend (instead of face-to-face classes), due to the pandemic. Most children and adolescent expressed their general unsatisfaction, choosing the “not at all” answer to the question “How much do you like attending online classes?” (41.9%). Out of the 155 answers given, only 16 of them expressed more positive views of the online school format (i.e., “I pretty much like them”—*N* = 11, 7.1%; “I like them a lot”—*N* = 5, 3.2%). Most children expressed their unsatisfaction due to the poor internet connection, which did not allow them to properly communicate and interact efficiently with their colleagues and teachers (i.e., 52, 41.93% answers out of 124). In comparison, others disliked online classes because they could not understand the contents of the lessons (22.58%), and the lack of face-to-face interaction with their colleagues and friends from school (19.35%). However, 15 answers positively described the advantages of online classes, through answers such as” I like online classes because it’s more comfortable to attend classes from home (*N* = 7), or “We are safe and protected at home” (*N* = 2) (see Table 5).

Concerning their self-reported coping mechanisms, i.e., personal resources and strategies used in these difficult pandemic times, most children and adolescents reported “positive thoughts” (*N* = 41, i.e., 27.70% answers out of a total of 148), followed by “family relationships” (14.19%), and protective equipment, such as masks, disinfectants, or gloves (around 10%). Similar coping mechanisms were perceived by the participants in others. For example, out of the 123 answers offered, 50 (40.65%) of them referred to positive thinking as the primary perceived coping mechanism used by other people to get through the pandemic (See Table 6). Other perceived resources were described as related to obeying the pandemic rules, such as social distancing (*N* = 18, 14.63%), and wearing a mask, and washing hands (12 answers, i.e., 9.75%).

Our final survey questions addressed the potential positive effects of the pandemic. Out of the 155 children and adolescents, 89 (57.4%) considered that the COVID-19 health crisis had no positive effects. However, 65 children considered that the pandemic had positive consequences, mostly related to time spent with their families (40.90%) (see Table 7).

## 3. Discussion

The present exploratory research investigated children and adolescents’ representation of the COVID-19 pandemic and their related emotions and cognitions. We also explored the self-reported protective factors and coping mechanisms, in addition to similar attributional emotional experiences, i.e., the ways participants evaluated others’ pandemic experiences. We aimed to use a mixed-method approach, including both qualitative and quantitative analyses, for a more comprehensive view of the pandemic’s impact. To our knowledge, the present study is the first to explore Romanian children’s and adolescents’ representation, emotions, and coping strategies related to the COVID-19 pandemic using a mixed-method, comprehensive approach.

Our primary findings suggested that most children and adolescents associated masks with the thought of the novel coronavirus, in line with previous similar research that suggested masks as the established symbol against COVID-19 [59] and “the ubiquitous symbol” of the pandemic [60]. The fact that masks (i.e., sanitary equipment/protection gear) are the most associated word/symbol with pandemic highlights may result from the priming effect of continuous messages related to the need to wear a mask to stop the virus, both in online and offline environments. For example, in Romanian cities, there are many street billboards picturing people wearing masks, in addition to the messages related to their importance (e.g., “Masks save lives!”). Furthermore, from public schools to private areas, any space people now enter highlights the need to wear a mask, or otherwise, access would not be permitted.

Furthermore, in the short time they went back to physical classes since the outbreak, all children and adolescents in our sample had to wear the mandatory mask to prevent getting infected or spreading the virus, if the case. Therefore, the emotional impact of the mandatory mask-wearing due to the significant related changes might have contributed to children’s and adolescents’ choice of pandemic representation. Future studies might continue this research idea by specifically exploring the emotions generated by face masks during and following the pandemic, preferably in experimental tasks in order to increase the findings’ generalizability.

We chose Ekman’s six basic emotions model due to its simplicity and focus on universally recognizable emotions. Our results indicated that the emotions most frequently associated with COVID-19 were sadness and fear. These findings align with previous results reported by Idoiaga Mondragonet et al. [23] and Casanova et al. [24], highlighting the potentially negative impact of the pandemic on the wellbeing of children. In addition, the participants reported a medium level of perceived adverse effects of the pandemic, motivated mainly by the transition from regular classes to the online school setting. Further research will be needed to understand better the impact of the pandemic on the wellbeing of children and establish whether the negative emotions reported by children in this context are severe enough to justify psychological intervention programs.

We also found a significant association between children’s self-reported levels of harmful effects of the pandemic and perceived adverse effects on their families. This specific result highlights the importance of the relationship between parents and children and how their children recognize, acknowledge, and understand parents’ emotional states. In a recent study, Maftei et al. [61] explored the link between children’s self-reported happiness and their parents’ perceived happiness, suggesting a significant, positive association between the two variables. More specifically, their study suggested that (1) the happier the parent (as perceived by the child), the happier the child; (2) the primary sources for children’s happiness were family and peer relationships. Therefore, our results align with these findings, emphasizing the symbiotic connection between parents and children’s emotional state. Moreover, in our sample, the children and adolescents reported positive thoughts and family relationships as their primary coping mechanisms during the pandemic, suggesting similar perceived coping mechanisms in the others around them. The practical implications of this specific result may be related to the need for parents’ emotional control to promote resilience and emotional equilibrium in their children.

Though initially intended as a short-term measure, online classes have become the norm in Romania and many countries worldwide. Starting March 2020, students attended online classes, and the general perception seems to be more harmful than positive. Most participants in our sample expressed their dissatisfaction concerning online school classes, primarily due to poor online interaction and the lack of face-to-face interaction with their peers. Though considerable efforts have been made to sustain an e-learning system that was much more rarely used in Romania before the pandemic (e.g., buying technological equipment for both students and their teachers; adapting the school curricula and final exams), and students generally accepted online learning and got used to it [9], it still seems unsatisfying and unattractive. These findings’ practical implications point to the need for investment in upgrading Internet connections so that e-learning works as intended. As suggested by our results, the poor Internet connection was the primary reason children and adolescents considered online classes unsatisfying. Considering that participants in our sample lived in urban areas, in Romania’s second-largest city, results reported by students living in rural areas or smaller cities might be even more pessimistic, given the less qualitative Internet connection in those areas.

Finally, more than half of the participants considered that the COVID-19 pandemic had no positive effects, while 40% considered the increased time spent with their families the primary positive consequences following the COVID-19 health crisis. These results are in line with those reported by Valadez et al. [27], who suggested that the most common answers related to the pandemic’s positive consequences, as perceived by adolescents, were related to spending time with their families. On the other hand, several researchers have suggested pandemic-driven post-traumatic growth [62,63]. which might also be the case for children and adolescents who perceive the pandemic as an opportunity for their families to strengthen their relationship. However, more than half of our participants did not consider any of the potential positive effects of the pandemic, and this emphasizes, once again, adolescents’ and children’s need for psychological support and assistance from both their families and specialists, as also suggested by previous studies [64,65,66].

Several limitations need to be mentioned concerning the present research. For example, the small number of participants in our sample lowers the generalizability of the present findings. Therefore, future studies might want to consider expanding the sample of participants by including more children and adolescents. Additionally, future studies might also want to consider a more heterogeneous sample in terms of age and living area, given that the urban versus rural living area seems to be highly important when discussing online classes’ perceptions and attitudes. For example, in Romania, rural areas have a significantly lower qualitative internet connection, which is a significant factor when assessing online classes’ quality and satisfaction.

Another important factor that we did not account for in our research was our participants’ economic status, which also plays a significant role in discussing the pandemic’s effects, perceptions, attitudes, or representations. Many people lost their jobs, and economic difficulties might have directly affected the children and adolescents in our sample. Future studies might also account for this variable and consider it in subsequent analyses. We also did not ask our participants about the potential casualties in their families (i.e., family members who died due to the COVID-19 disease), as this might be highly significant when exploring our data. However, the potential priming effects should be controlled for when future studies should explore this variable. Additionally, our study was cross-sectional, and future experimental approaches would increase the generalizability of the findings. Finally, the scale we used was not previously validated on a similar sample. Though we pretested it prior to the study and reported no issues, other supplementary studies are needed to assess the reliability of the quantitative items.

Despite these limitations, we consider that the findings of our exploratory, mixed-method approach suggest a potentially significant contribution to the theoretical framework assessing the COVID-19 psychological impact upon children and adolescents, as well as a potentially important source of information for potential intervention programs, aiming to increase both the psychological wellbeing of children and adolescents, as well as the quality of the Romanian e-learning system.

## Figures and Tables

**Table 1:** Participants’ answer to Question 1: “When you think about the COVID-19 pandemic, which are the first three words that come to your mind?”.

Participants’ Exact Answers	*N*	*%*
Pandemic	20	14.53
Disease	13	9.93
Mask	57	41.3
Doctors	3	2.17
Disinfectant	6	4.65
Danger	1	0.07
Quarantine	4	2.89
Virus	12	8.95
Sadness	3	2.17
Something that seems never to end	2	1.44
Keep moving no matter what	1	0.07
Death	7	5.32
Family	3	2.17
Online	3	2.17
Isolation	3	2.17

N_answers coded_ = 138.

**Table 2:** Self-reported and perceived adverse effects of the COVID-19 pandemic (*N* = 155).

Self-Reported Negative Effects	*N*	*%*
1 (not at all)	15	9.7
2 (a little)	40	25.8
3 (moderately)	42	27.1
4 (pretty much)	31	20.0
5 (a lot)	27	17.4
Perceived negative effects on their families		
1 (not at all)	18	11.6
2 (a little)	33	21.3
3 (moderately)	43	27.7
4 (pretty much)	46	29.7
5 (a lot)	15	9.7

**Table 3:** Source of the self-perceived adverse effects of the COVID-19 pandemic.

Category	Example	*N*	%
New health rules and regulations	Since the pandemic, we have to wear a mask all the time	11	12.15
Online classes	The worst thing about the pandemic is the online classes	24	26.37
Lack of face-to-face interaction	I could not see and talk to my friends and colleagues without a computer	12	13.27
Negative emotions	Since the pandemic, I have been sad/worried a lot	13	13.98
Isolation	I felt isolated from everything	11	12.15
Change	I can’t handle any more changes	7	7.75
Lack of physical contact	Even if we are back to school from time to time, I can’t hug or touch my friends anymore; it’s against the rules.	7	7.75
Self or others’ health issues	I think about all the people who died or got sick I constantly think about the time I was sick, when I had COVID-19	4	4.39
School-related issues	I am not doing good in school anymore, since the pandemic, I can’t concentrate as much as before COVID-19.	2	2.19

*N*_answers coded_ = 91.

**Table 4:** Source of others’ perceived adverse effects of the COVID-19 pandemic.

Category	Example	*N*	%
New rules for work/employment	Because my parents had to work from home, and they did not like that at all.	4	6.25
Online classes	My family didn’t find it easy to adjust to my online classes	5	7.50
Lack of face-to-face interaction	My parents missed seeing their friends and family face to face	2	3.34
Negative emotions	We were all worried and scared because of the virus	6	8.80
Isolation	We all felt isolated inside our homes	5	7.50
Change	It is hard to follow the new rules for shopping, for example, such as specific shopping hours/They had to make a lot of changes and adapt to new rules	3	4.95
Direct experience of COVID-19	Because my parents had COVID-19.	3	4.95
Health regulations	They did not like wearing a mask all the time2	6	8.80
Worries related to others’ health issues (e.g., grandparents)	My parents felt worried all the time for my grandparents; they were afraid they might catch the COVID-19 virus and die.	13	20.31
Parents’ worries related to their children	My parents were worried for me and my brother, that we would get sick.	4	6.25
Worries about personal health issues	My parents were worried they would get sick and die.	3	4.95
Economical issues	We ran out of money/My parents were worried we would not have money for food.	10	16.40

N_answers coded_ = 64.

**Table 5:** Perception of the online classes during the COVID-19 pandemic.

Positive Perception Source	Example	*N*	%
Poor Internet connection	The connection is really bad, half of the time we’re just trying to reconnect.	52	41.93
Lack of understanding the educational content	I can not understand everything the teachers as saying or trying to teach us.	28	22.58
Lack of face-to-face interaction with their colleagues and friends from school	I can’t see my friends and colleagues from school anymore	24	19.35
Boredom	Online classes are really boring, and I get bored a lot.	3	2.41
Tiredness	Online classes tire me/I feel extremely tired after attending the online classes.	2	1.61
Negative perception source	Example	*N*	%
Feelings of security/protection	We are safe and protected at home.	2	1.61
Lower complexity	It’s easier to learn online, not as hard as it is in school.	4	3.22
Comfort	It’s much more comfortable to learn from your own home.	7	5.68
Lack of protective equipment	We do not have to wear a mask at home, and I hate masks.	2	1.61

*N*_answers coded_ = 124.

**Table 6:** Perception of self-reported and others’ perceived coping mechanisms during the COVID-19 pandemic.

Self-Reported Coping Mechanisms (Answers Coded: *N* = 148)	Example	*N*	%
Positive thinking	I always thought about good, positive things instead of the pandemic.	41	27.70
Family relationships	Being close to my family helped me the most.	21	14.19
Using protective equipment	Since the pandemic started, I wear a mask all the time, and I always wash my hands so I don’t get sick	15	10.14
Optimism	I remained optimistic: it has to end at a certain point, and things will be better.	9	6.09
Art/Artistic activities	I started painting and drawing a lot; it helped me especially during the quarantine.	7	4.8
Faith	Praying and talking to God helped me a lot.	3	2.03
Technology	The pandemic was a lot easier to handle due to technology, it personally helped me a lot with everything.	3	2.03
Emotion regulation	Staying calm even when I was really scared.	2	1.36
Sports	I played a lot of football and basketball, anything to keep me moving	4	2.71
Cognitive suppression	Thinking about anything else but the pandemic	14	9.46
Play	I played a lot and it helped me	6	4.06
Obeying the social distancing rules	We stayed inside our home as much as possible to respect the social distancing rules	12	8.11
Friends	My friends helped me a lot, especially at the beginning, when I was so scared!	11	7.32
Others’ coping mechanisms (Answers coded: *N* = 123)	Example	*N*	%
Positive thinking		50	40.65
Cognitive suppression	Thinking about anything else but the pandemic	10	8.13
Optimism	I remained optimistic: it has to end at a certain point, and things will be better.	9	7.31
Family relationships	I think their family helped them the most, being close to one another.	9	7.31
Technology	Technology helped people the most because it helped all of us communicate.	1	0.81
Pets	I think it was important for people to have a pet during the pandemic, it might have helped them a lot.	2	1.63
Unknown source	I don’t know	4	3.27
Self-trust	Believing in themselves	2	1.63
Friends	Friends are always very helpful; they helped me, so I think other people were helped by friends, too.	2	1.63
Entertainment	Playing games and watching TV	3	2.44
Obeying the rules	Respecting the social distancing rules in shops and everywhere else	18	14.63
Using protective equipment	Wearing a mask and gloves	12	9.75
Positive emotions	I think that people who tried to feel happy got through the pandemic easier	1	0.81

**Table 7:** Perception of the potential positive COVID-19 pandemic outcomes.

Positive Outcomes	Example	N	%
Evolution	The new technological changes help as evolve	1	4.54
Family time	The pandemic helped us spend more time with our families	9	40.90
Social distance	I am happy that, due to the pandemic, I did not have to socialize with others anymore	1	4.54
Environmental improvements	The pandemic will reduce global warming and pollution	2	9.09
Self-care improvement	The pandemic taught us to take more care of ourselves	3	13.64
Responsibility	The pandemic made us more responsible	4	18.20
Healthcare improvements	The pandemic made us stronger; we do not get sick as quickly as before	2	9.09

N_answers coded_ = 22.

## Data Availability

The raw data supporting this article’s conclusions will be made freely available by the authors upon reasonable request.

## Appendix A

The following items refer to the current COVID-19 pandemic. Please carefully read each item and answer the questions below. There are no right or wrong answers.

1. When you think about the COVID-19 pandemic, which are the first three words that come to your mind?

a.

b.

c.

2. When you think about the COVID-19 pandemic, which is the emotion you usually feel?

a. Happiness

b. Sadness

c. Surprise

d. Fear

e. Anger

f. Disgust

g. I don’t know

3. We often hear about the negative consequences of the current pandemic. However, these adverse effects may not be felt by everyone, or they might feel different for each person. On a scale ranging from 1 (not at all) to 5 (very much), how much did the pandemic negatively affect you?

1. not at all

2. a little bit

3. moderately

4. pretty much

5. very much

4. Why? (Please explain your answer to question 3)

5. We often hear about the negative consequences of the current pandemic. However, these adverse effects may not be felt by everyone, or they might feel different for each person. On a scale ranging from 1 (not at all) to 5 (very much), how much did the pandemic negatively affect your family?

a. not at all

b. a little bit

c. moderately

d. pretty much

e. very much

6. Why? (Please explain your answer to question 5)

7. Due to the pandemic, classes had to be held online, in most of the past few months. On a scale ranging from 1 (not at all) to 5 (very much), how much do you like attending online classes?

1. not at all

2. a little bit

3. moderately

4. pretty much

5. very much

8. Why? (Please explain your answer to question 7)

9. Which are, in your opinion, the things that helped you the most to cope with and get through the changes and the general context brought by the pandemic?

10. Which are, in your opinion, the things that helped other people the most to cope with and get through the changes and the general context brought by the pandemic?

11. In your opinion, are there any positive sides or effects of the COVID-19?

12. Why? (Please explain your answer to question 11)
